# EARLY FRAILTY PHENOTYPES AND PREDICTIONS OF COGNITIVE AGING: EVIDENCE FROM THE VICTORIA LONGITUDINAL STUDY

**DOI:** 10.1093/geroni/igz038.1077

**Published:** 2019-11-08

**Authors:** Linzy Bohn, Yao Zheng, G Peggy McFall, Roger A Dixon

**Affiliations:** University of Alberta, Edmonton, Alberta, Canada

## Abstract

Frailty is an aging condition that reflects multisystem decline. A prominent approach to frailty assessment is to create an index, whereby responses across multiple indicators of aging systems are summed to create a single score. These studies indicate that frailty is associated with adverse aging outcomes (e.g., mortality, dementia). We employ a data-driven approach to detecting and differentiating emerging frailty phenotypes and examine their associations with non-demented cognitive aging trajectories. Participants (n = 653; M age = 70.6, range 53-95) were community-dwelling older adults from the Victoria Longitudinal Study. Participants contributed (a) baseline data for 30 frailty-related items representing deficits across 7 domains (e.g., instrumental and cardiovascular health) and (b) longitudinal data for latent variables of executive function, speed, and memory. For each participant, we calculated the proportion of deficits present in each frailty-related domain and submitted these data to a latent profile analysis (LPA; Mplus 7.0). We used latent growth modeling (LGM) to test these frailty phenotypes for prediction of cognitive performance and decline. LPA results revealed three profiles, one large normal low-frailty profile and two emerging frailty phenotypes. Whereas the latter represented profiles of individuals with respiratory-type frailty (i.e., marked impairment in respiratory function; 7%) and mobility-type frailty (i.e., marked impairment in mobility function; 9%), the former featured limited impairment across frailty domains (83%). Findings from LGM indicated that these profiles were differentially related to cognitive performance and decline. Data-driven approaches can help detect early differentiation of frailty profiles and contribute to personalized intervention.

